# Protective Effect of Lactobacillus rhamnosus GG and its Supernatant against Myocardial Dysfunction in Obese Mice Exposed to Intermittent Hypoxia is Associated with the Activation of Nrf2 Pathway

**DOI:** 10.7150/ijbs.36465

**Published:** 2019-09-07

**Authors:** Hui Xu, Jiqun Wang, Jun Cai, Wenke Feng, Yonggang Wang, Quan Liu, Lu Cai

**Affiliations:** 1 Cardiovascular Center, the First Hospital of Jilin University, Changchun, 130021 China; 2 Pediatric Research Institute, Department of Pediatrics, the University of Louisville, Norton Healthcare, Louisville, KY 40202, USA; 3 Department of Pharmacology and Toxicology, the University of Louisville School of Medicine, Louisville, KY 40202, USA; 4 Division of Gastroenterology, Department of Medicine, the University of Louisville School of Medicine, Louisville, KY 40202, USA

**Keywords:** intermittent hypoxia, cardiomyopathy, probiotics, inflammation, oxidative stress, nuclear factor erythroid 2-related factor 2

## Abstract

Prolonged intermittent hypoxia (IH) has been shown to impair myocardial function (mainly via oxidative stress and inflammation) and modify gut microbiota in mice. Gut microbiota plays an important role in health and disease, including obesity and cardiovascular disease (CVD). Probiotics refer to live microorganisms that confer health benefits on the host after administration in adequate amounts. Research on novel probiotics related therapies has evoked much attention. In our previous study, both Lactobacillus rhamnosus GG (LGG) and LGG cell-free supernatant (LGGs) were found to protect against alcohol-induced liver injury and steatosis; however, the effects of LGG and LGGs on cardiac tissues of obese mice exposed to IH have not been determined. Here we exposed high-fat high-fructose diet (HFHFD)-induced obese mice to IH, to establish a model of obesity with obstructive sleep apnea (OSA). Mice were divided into four groups: (1) HFHFD for 15 weeks; (2) HFHFD for 15 weeks with IH in the last 12 weeks (HFHFD/IH); (3) and (4) HFHFD/IH plus oral administration of either LGG (10^9^ CFU bacteria/day) or LGGs (dose equivalent to 10^9^ CFU bacteria/day) over the 15 weeks, respectively. Compared to HFHFD mice, HFHFD/IH-mice showed heart dysfunction with significant cardiac remodeling and inflammation; all these pathological and functional alterations were prevented by treatment with both LGG and LGGs (no significant difference between LGG and LGGs in this respect). The cardioprotective effect of LGG and LGGs against IH/HFHFD was associated with up-regulation of nuclear factor erythroid 2-related factor 2(Nrf2)-mediated antioxidant pathways. Our findings suggest a cardioprotective effect of LGG and LGGs in obese mice with OSA.

## Introduction

Obstructive sleep apnea (OSA) is a common disease characterized by recurrent upper airway obstruction during sleep that results in intermittent hypoxia (IH) 1. OSA is associated with increased cardiovascular morbidity and mortality 2. An estimated 60%-90% patients with OSA are obese, and obesity is the predominant underlying risk factor for metabolic syndrome 3, 4. Additionally, obesity is one of the major cardiovascular disease (CVD) risk factors associated with OSA 5. Effective treatment of OSA may significantly decrease mortality 6. IH is believed to play an important role in the morbid consequences of OSA on multiple organs, most likely via enhanced oxidative stress and inflammation.

Nuclear factor erythroid 2-related factor 2 (Nrf2) was shown to function as an antioxidant regulator in CVD that are associated with oxidative stress 7. Under normal circumstances, Nrf2 is anchored to Keap1 in the cytoplasm, and the Nrf2-Keap1 complex facilitates degradation of Nrf2 to help maintain the intracellular Nrf2 level 8. Once exposed to increasing cellular oxidants, Nrf2 dissociates from Keap1 and translocates to the nucleus, where it binds to an antioxidant response element (ARE) and initiates transcription of antioxidant genes 9; these genes encode a variety of cardioprotective proteins such as heme oxygenase 1 (HO-1), quinone oxidoreductase 1 (NQO1), and catalase (CAT) 10. In our previous study, IH was shown to downregulate Nrf2 expression, while sulforaphane was shown to restore Nrf2 expression and initiate its downstream genes in cardiac tissue 11. Therefore, up-regulation of Nrf2 may be an effective approach to protect the heart from IH, which is a key pathological characteristic of OSA.

In addition, increasing evidence suggests that OSA is also involved in the development of metabolic syndrome and its associated cardiovascular outcomes. For instance, exposure to IH may alter the gut ecology including the microbiome and metabolites 12. In the setting of gut dysbiosis, microbiota may contribute to various cardiometabolic consequences, such as type 2 diabetes, hepatic steatosis, hypertension, and atherosclerosis 13, 14. Microbial sequencing analysis has unraveled a wealth of evidence about the characteristic gut microbiota associated with CVD 15, 16. The potential mechanisms linking gut microbiota to CVD are complex; these include the direct immunomodulatory effects of bacteria and their products, as well as the effects of microbial metabolites on thrombosis and development of atherosclerosis 17. Some biologically active metabolites produced by gut microbiota can be absorbed into the systemic circulation; subsequently, these are metabolized by host enzymes and serve as mediators of gut metabolic effects in the host 18. Thus, the gut microbiota functions as a virtual endocrine system that communicates with distal organs through metabolism related pathways. The myriad linkages between gut dysbiosis and CVD susceptibility have drawn a spotlight on the gut microbiome and microbial metabolites as potential novel therapeutic targets.

The term probiotics refers to live microorganisms that are able to affect the diversity and the composition of the gut microbiota. Administration of probiotics in adequate amounts has been shown to confer health benefits 19. Additionally, probiotics have been shown to exhibit favorable effects on metabolic function in animal models of metabolic syndrome 20. In a small double-blind, placebo-controlled study, patients with stable heart failure were randomly assigned to placebo or probiotic treatment groups for 3 months; the results showed significant improvement in both left atrial diameter and ejection fraction in the probiotic group^21^. Butyrate released by *Faecalibacterium prausnitzii* in the culture supernatant was shown to modulate T cells and exert an anti-inflammatory effect in the setting of inflammatory bowel disease 22; this suggests a potential therapeutic effect of microbial metabolites. Additionally, we previously demonstrated a protective effect of lactobacillus rhamnosus GG (LGG) cell-free supernatants (LGGs) against acute-alcohol-induced hepatic steatosis and injury 23. However, it remains unknown whether probiotics such as LGG or their supernatant can activate Nrf2 in the heart to protect from oxidative stress and damage.

In the present study, we focused on the potential role of modulation of gut microbiota as a novel therapeutic method with particular emphasis on the interaction between probiotics, their metabolites, and IH-induced cardiomyopathy in an animal model of obesity. We established a high-fat high-fructose diet (HFHFD) fed mice model exposed to IH to mimic OSA and investigated the effect of oral supplementation of *LGG* (the most common probiotic available) and LGGs. We found that supplementation with LGG or LGGs improved myocardial function and attenuated inflammation and oxidative stress in obese mice with IH-induced cardiomyopathy. In addition, we demonstrate that LGG and its metabolites in supernatant are potential activators of Nrf2 that trigger Nrf2-dependent antioxidative response in cardiac tissue.

## Material and methods

### Preparation and administration of LGG or LGGs

LGG obtained from American Type Culture Collection (ATCC 53103, Rockville, MD) was cultured in MRS broth (BD Biosciences-Advanced Bioprocessing, Sparks, MD) at 37°C in accordance with ATCC guidelines. Probiotics were harvested form MRS broth by centrifugation, and colony forming units (CFU) were counted by dilution and streaking on MRS agar plates (Difco) at 37°C overnight; finally, we used a bacterial density of 10^9^ CFU/mL. To prepare supernatant, culture broth was centrifuged and filtered through 0.22 μm filters when the bacterial density reached 10^9^CFU/mL 23. The bacterium and supernatant were stored at 4°C and orally administered to mice within a week of preparation.

### Animals

Six-week-old C57BL/6J male mice obtained from Jackson Laboratory (Bar Harbor, ME) were randomly assigned to four groups. All mice were fed a HFHFD (D12450JL, Research Diets, New Brunswick NJ) for 15 weeks. These mice were divided into four groups: (1) HFHFD group; (2) HFHFD with exposure to IH during the last 12 weeks (HFHFD/IH group); (3) and (4) HFHFD/IH with oral administration of LGG (10^9^ CFU bacteria/day) or LGGs (dose equivalent to 10^9^ CFU bacteria/day) over the entire experiment (HFHFD/LGG/IH and HFHFD/LGGs/IH groups, respectively). The procedure for exposure to IH is described elsewhere^24^. Briefly, the IH paradigm comprises of alternating cycles of 20.9% O_2_ /8% O_2_ FiO_2_ (30 episodes per hour) with 20s at the nadir FiO_2_ during the 12 h light phase. All animal procedures were approved by the Institutional Animal Care and Use Committee (IACUC) of the University of Louisville, which is certified by the American Association for Accreditation of Laboratory Animal Care.

### Echocardiography

All mice underwent transthoracic echocardiography (echo) for assessment of cardiac function, as described elsewhere 25. Briefly, a high-resolution imaging system (Vevo 770, Visual Sonics, Canada) equipped with a high-frequency RMV 707B ultrasound probe was used to examine isoflurane anesthetized mice. Interventricular septum (IVS), left ventricular (LV) internal dimension (LVID), and LV posterior wall (LVPW) were measured from LV M-Mode images. Fractional shortening (FS), ejection fraction (EF), and LV mass were acquired by Vevo770 software.

### Histological staining

10% buffered formalin fixed cardiac specimens were processed in gradient alcohol, xylene, and embedded in paraffin. Subsequently, paraffin blocks were sectioned at 5μm thickness. The sections were stained with hematoxylin and eosin (HE) after rehydration. To detect collagen deposition and fibrosis in cardiac tissues, sections were stained with Picro-Sirius Red. Cardiac frozen sections were fixed in acetone for 15-20 min and stained with FITC-conjugated wheat germ agglutinin (Invitrogen) to determine myocyte size. Microscopic images were captured with Nikon ECLIPSE E600. The quantitative results were analyzed using the ImageJ software.

### Immunohistochemistry and immunofluorescence

Methods for immunohistochemical staining are described elsewhere 26. Antibodies used for IHC were fibronectin (Abcam), collagen1A1 (Santa Cruz), TGF-β (Abcam), and IL-1β (Santa Cruz). Primary antibody labeling detection was performed after incubation with the appropriate DAB incubated secondary antibody. For fluorescent labeling of Nrf2 nucleus translocation, frozen sections were fixed with 4% PFA for 10 min and permeabilized with 0.1% Triton X-100 for 20 min. After blocking with 5% BSA for 1h, sections were stained with Nrf2 antibody (Abcam), and then stained with secondary antibody, Texas Red (Invitrogen). Nuclei were counterstained with hematoxylin in IHC experiments and with DAPI in SlowFade® Gold Anti-fade Mountant in IF experiments.

### Western blotting

Cardiac tissues were homogenized with RIPA lysis buffer to prepare lysates. Protein samples were subjected to SDS-PAGE, and transferred onto a PVDF membrane (Bio-Rad, Hercules, CA). After blockade with 5% skimmed milk, membranes were incubated with antibodies. Primary antibodies to fibronectin, CTGF, TNF-α, 3-NT, 4-HNE, Nrf2, and HO-1 were obtained from Abcam (Cambridge, MA); collagen1A1, ANP, IL-1β, NQO1, CAT, SOD2, and β-actin were obtained from Santa Cruz Biotechnology (Santa, CA); PAI-1 was obtained from BD Biosciences (Franklin Lakes, NJ); phosphorylated NF-κB and total NF-κB, as well as horseradish peroxidase (HRP)-conjugated anti-mouse and anti-rabbit secondary antibodies were obtained from Cell Signaling Technology (Danvers, MA). Western blot images were acquired using ChemiDoc Touch Imaging System (Bio-Rad). Grayscale values of bands were analyzed using the Image Lab software (Bio-Rad); protein expressions were normalized relative to those of β-actin.

### Quantitative real-time PCR

mRNA was extracted from cardiac tissues using Trizol (Invitrogen). The RNA purity and concentration was quantified using the Nano Drop ND-1000 spectrophotometer. cDNA was synthesized from 1 μg total RNA using AMV transcriptase 5×buffer. Quantitative PCR was performed with TagMan Universal PCR master mix (Invitrogen) using the LightCycler 96 RT-PCR system (Roche Diagnostics Corporation, Indianapolis, IN). Primers against β-MHC (Mm00600555_ml), Hmox1 (Mm00516005_m1), NQO1 (Mm01253561_m1), CAT (Mm00437992_m1), Adipor1 (Mm01291334_mH), Adipor2 (Mm01184032_m1) and GAPDH (Mm99999915_gl) were obtained from Thermo Fisher (Grand Island, NY). To determine fold differences between samples, comparative cycle time (C_t_) values were used for analysis, and all samples were normalized to GAPDH mRNA levels.

### Statistical analysis

Data are presented as mean ± standard deviation (SD). Between-group differences were assessed using one-way ANOVA, followed by post-hoc pairwise repetitive comparisons with Turkey test using Graphpad Prism version 6.0c.

## Results

### LGG or LGGs prevented IH-induced morphological changes and cardiac dysfunction

Echo analysis revealed that the structural parameters such as LV internal systolic diameter (LVID; s) and LV end systolic volume (LV vol; s) of IH-exposed obese mice were significantly higher than those of obese mice without IH. This increase was prevented in animals that were administered LGG. Analysis of functional parameters showed that EF% and FS% in the IH group were significantly lower than that in the normoxic group. After LGG administration, the EF% and FS% were comparable to those in the normoxic group, which was indicative of protective cardiac remodeling. The above parameters showed obvious changes after administration of LGGs although these were not statistically significant; however, there was a statistically significant improvement in EF% (Table 1).

**Figure 1:**
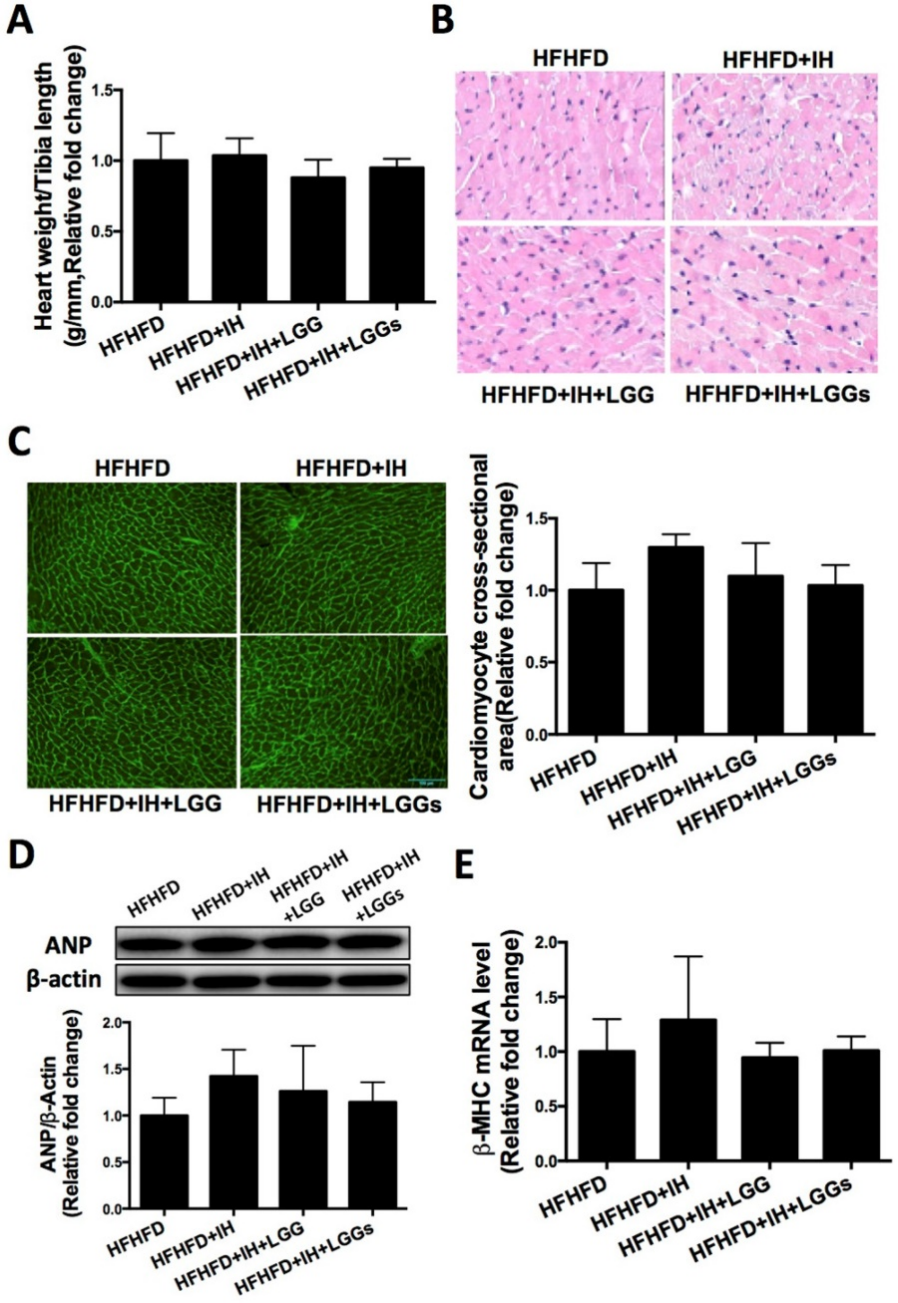
** General feature and cardiomyocyte size was not obviously altered by IH or supplementation with LGG or LGGs. A,** Weight of heart to tibia length after exposure to IH for 12 weeks. **B,** HE staining of heart sections reveal disorganized structure in IH group, whereas treatment with LGG or LGGs significantly inhibited IH-induced disorganization of cardiac tissue. **C,** Wheat germ agglutinin (green)-stained cardiac cross-sections with quantification of mean cardiomyocyte cross-sectional area relative fold change. **D,** Western blot from cardiac extracts using antibody against ANP. β-actin was used as loading control. Quantitative analysis showing ANP expression relative to that of β-actin. **E,** mRNA level of β-MHC in IH-treated mice heart after administration of LGG or LGGs. All bar graphs are plotted as mean± SD. *P<0.05, **P<0.01 vs. HFHFD+IH group.

In obese mice, exposure to IH for 12 weeks did not affect the heart weight-to-tibia length ratio. Treatment with LGG or LGGs also did not affect the heart weight-to-tibia length ratio (Fig. 1A). IH-induced morphological changes in obese mice were assessed by HE staining; IH induced an increase in zones of structural disorganization in the myocardium; however, this change was not observed in mice in the LGG or LGGs groups (Fig. 1B).

### LGG and LGGs did not affect IH-induced changes in cardiomyocyte size and cardiac wall thickness

To compare the size of cardiomyocytes, heart sections were stained with wheat germ agglutinin (WGA)-conjugated with FITC (green). The cross-sectional area of cardiomyocytes was calculated by measuring WGA-enclosed areas. The mean size of cardiomyocytes was slightly increased; however, there was no significant difference between IH/obese mice and obese mice. Moreover, there was no significant change in the groups treated with either LGG or LGGs (Fig. 1C). Echo parameters, such as left ventricular posterior wall (LVPW) and interventricular septum (IVS) were also consistent with findings of WGA staining (Table 1).

We analyzed the effects of IH and LGG or LGGs on the expressions of hypertrophy related genes. Expressions of ANP protein (Fig. 1D) and β-MHC mRNA (Fig. 1E) were not significantly changed in response to IH under obese conditions independent of treatment with LGG or LGGs. These data indicate that IH did not aggravate hypertrophy under obese conditions, and that LGG or LGGs treatment also had no significant effect on cardiac wall thickness or cardiomyocyte size in IH/obese mice.

### LGG or LGGs exerted anti-fibrotic effect on the hearts induced by IH in obese mice

**Figure 2:**
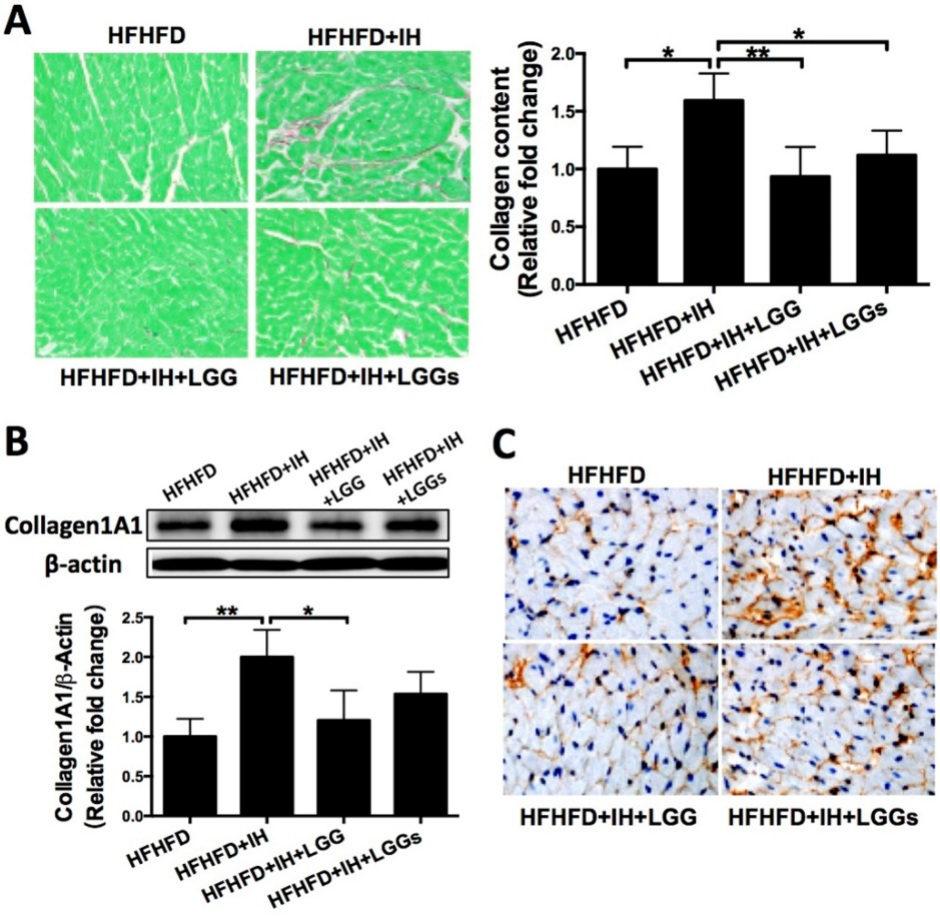
** Administration of LGG or LGGs reduced cardiac collagen accumulation in IH mice. A**, Picro-Sirius Red staining of heart sections showing extensive fibrotic areas in the IH group. Quantitative analysis shows that LGG or LGGs treatment significantly inhibited IH-induced fibrosis in cardiac tissue. **B**, Western blot depicting pro-fibrotic protein expression of Collagen1A1 in cardiac tissues. β-actin used as loading control. Quantitative analysis showing Collagen1A1 expression relative to that of β-actin. **C**, Representative histochemical staining of Collagen1A1 in heart sections. All bar graphs are plotted as mean± SD. *P<0.05, **P<0.01, ***P<0.001 vs. HFHFD+IH group.

**Figure 3:**
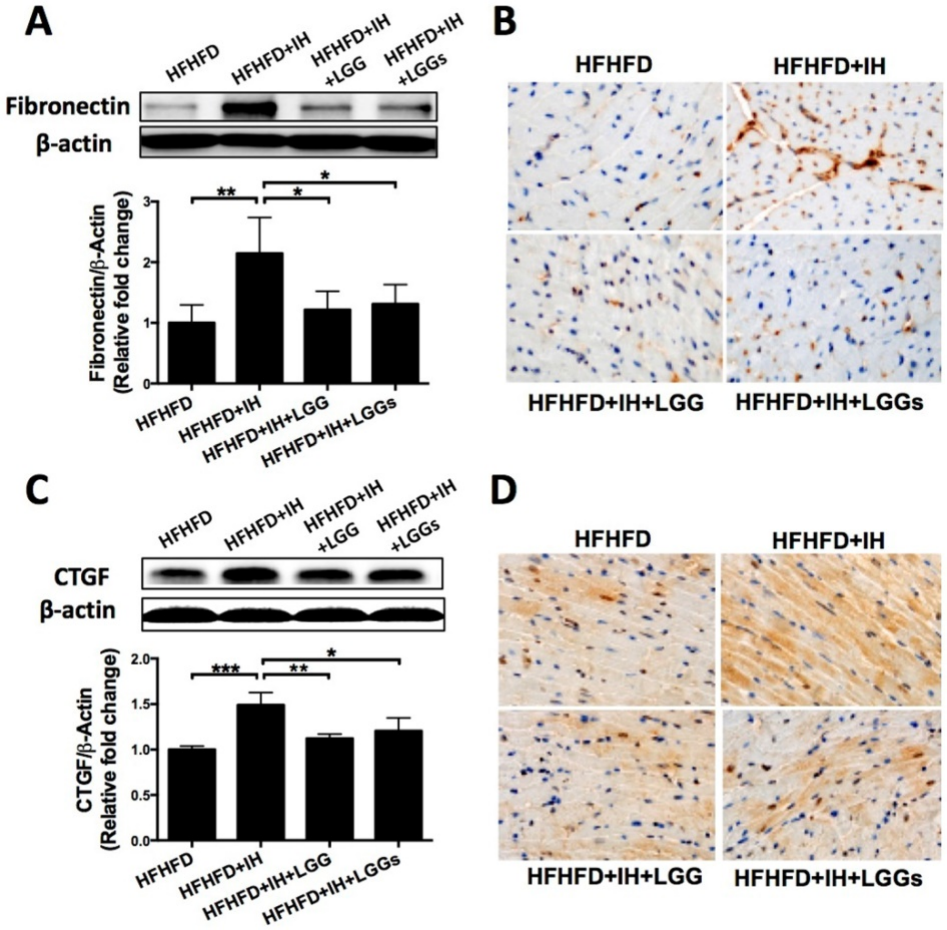
** Administration of LGG or LGGs reduced pro-fibrotic markers in IH mice heart. A, C,** Western blot depicting pro-fibrotic protein expressions of fibronectin and CTGF in cardiac tissues. β-actin used as loading control. Quantitative analysis showing expressions of fibronectin and CTGF relative to that of β-actin. **B, D,** Representative immunohistochemical staining of fibronectin and TGF-β in heart sections. All bar graphs are plotted as mean± SD. *P<0.05, **P<0.01vs. HFHFD+IH group.

Picro-Sirius Red staining revealed greater interstitial collagen deposition between cardiomyocytes of IH/obese mice as compared to that in obese mice. Cardiac collagen deposition in the LGG and LGGs groups was similar to that in the normoxia group, which suggested a protective effect of LGG or LGGs on IH-induced fibrotic responses (Fig. 2A). Western blotting analysis with the total protein lysates isolated from the heart revealed an increase in the protein levels of pro-fibrotic markers (such as collagen1A1, fibronectin, and CTGF) in IH/obese mice compared to obese mice. Treatment of IH/obese mice with LGG or LGGs prevented the effect of IH exposure on these fibrotic responses (Fig. 2B, Fig. 3A, C).

We performed immunohistochemistry to confirm the protective effect of LGG and LGGs against IH-induced fibrosis. Treatment with LGG and LGGs was associated with decreased recovery of collagen1A1 (Fig. 2C), fibronectin (Fig. 3B) and TGF-β (Fig. 3D). Collectively, administration of LGG and LGGs significantly prevented IH-related cardiac remodeling. In general, the anti-fibrotic effect of LGG on the heart of IH/obese mice seemed somewhat better than that of LGGs, although there was no significant difference in this respect (Figs. 2 and 3).

### LGG or LGGs significantly inhibited IH-mediated cardiac inflammation in obese mice

**Figure 4:**
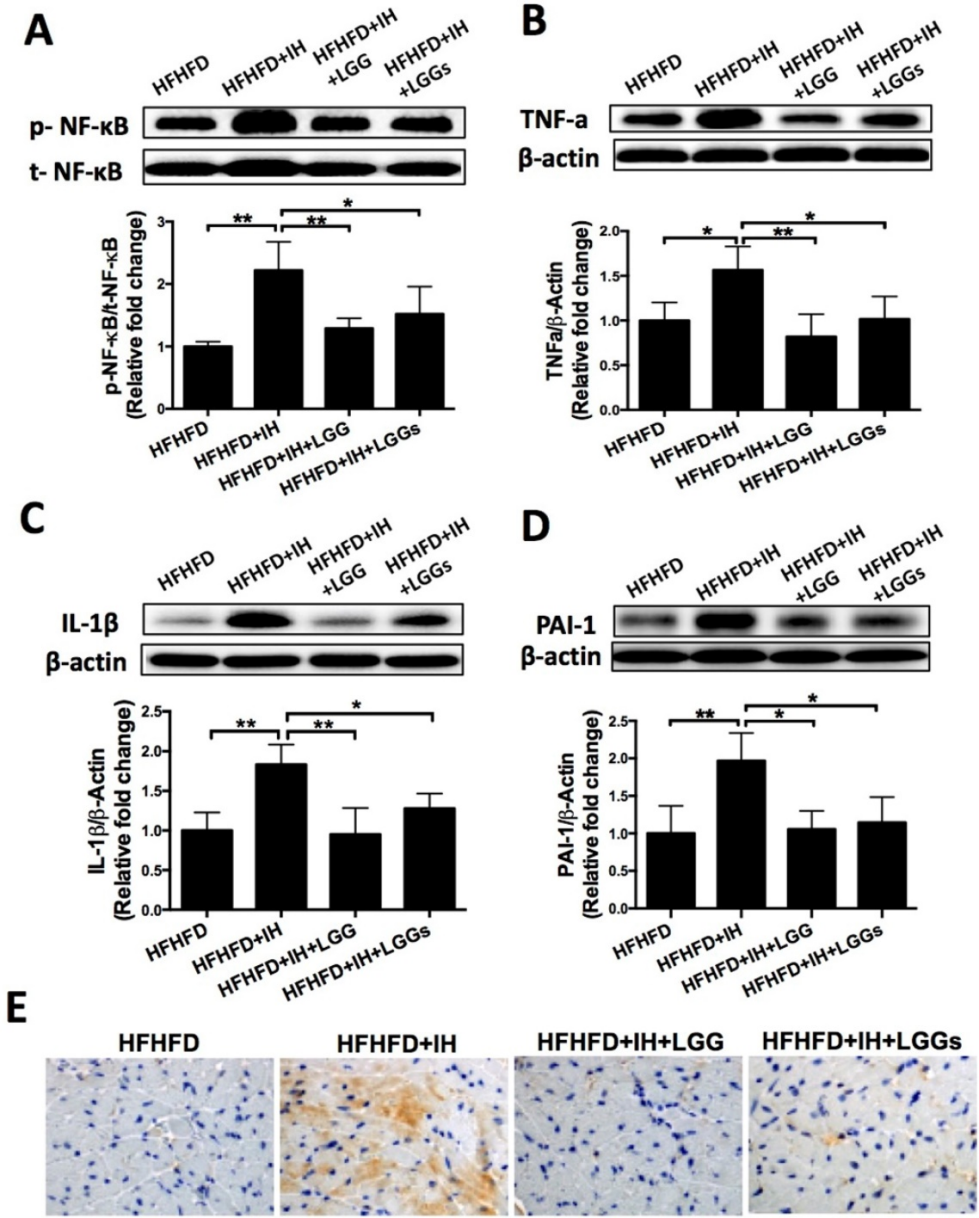
** LGG and LGGs reduced inflammation responses in IH mice hearts. A, B, C, D**, Western blot depicting pro-inflammatory protein expressions of p-NF-κB, TNF-a, IL-1β, and PAI-1 in cardiac tissues. β-actin used as loading control. Quantitative analysis showing expressions of p-NF-κB, TNF-a, IL-1β, and PAI-1 relative to that of β-actin. **E**, Representative photomicrographs of heart sections stained with antibody against IL-1β (brown staining) and hematoxylin. Graphs show inflammatory area stained positive for IL-1β. All bar graphs are plotted as mean± SD. *P<0.05, **P<0.01 vs. HFHFD+IH group.

Phosphorylation of NF-κB is a critical event that links signals from hypoxic stress with downstream effectors of inflammation 27. Western blotting of phosphorylated NF-κB (an inflammatory marker) with cardiac protein lysates showed that IH significantly increased phosphorylation level of NF-κB; this effect was significantly prevented by treatment with LGG or LGGs (Fig. 4A). Immunoblot analysis revealed that that exposure of obese mice to IH significantly increased the protein expressions of pro-inflammatory markers TNF-α, IL-1β, and PAI-1; these changes were significantly prevented by treatment with LGG or LGGs (Fig. 4B-D).

The anti-inflammatory effects of LGG and LGGs were further corroborated by the significantly higher expression of IL-1β in the cardiac tissues of IH/obese mice as compared to obese mice (Fig. 4E). However, this adverse effect was significantly prevented by treatment with LGG or LGGs; there was no difference between LGG and LGGs in this respect (Fig. 4E). The above results suggested that administration of LGG or LGGs negatively regulated inflammation in hearts exposed to IH.

### The antioxidant effect of LGG or LGGs on IH-induced oxidative stress and damage was associated with activation of Nrf2 pathway

**Figure 5:**
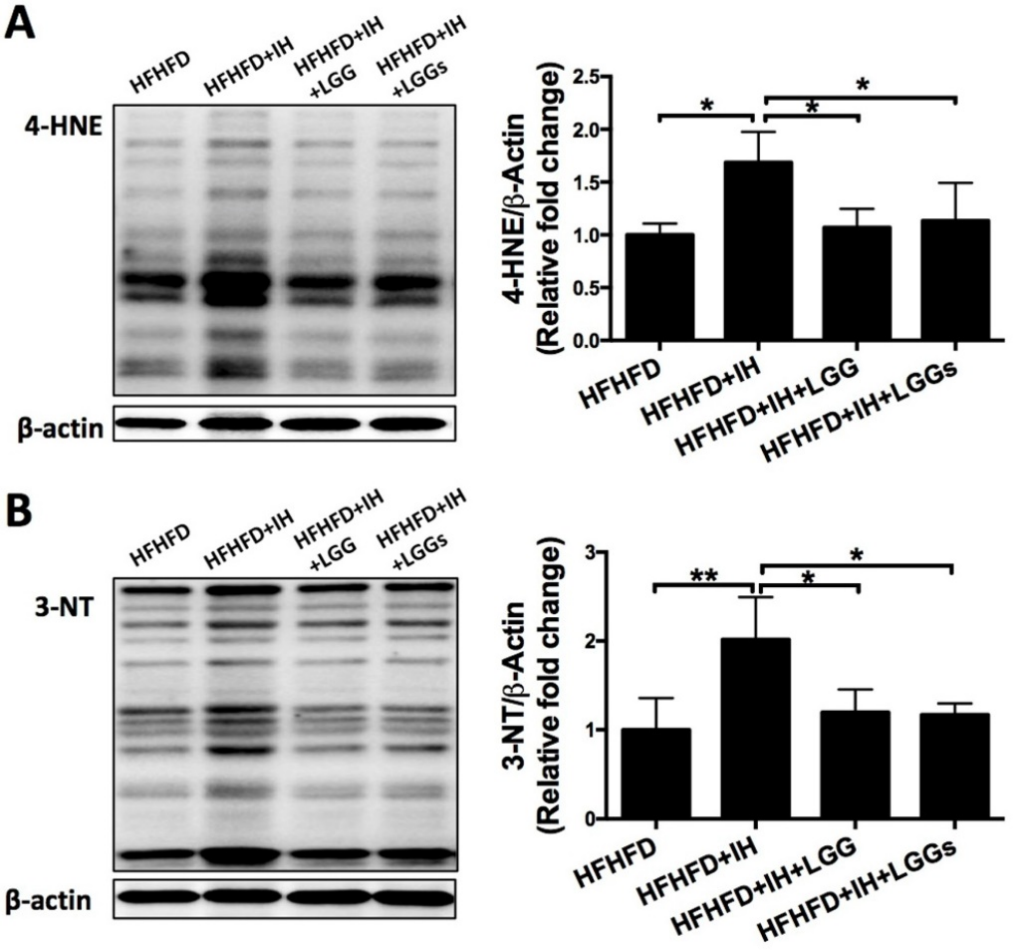
** IH mice treated with LGG and LGGs showed reduced oxidative stress damage in hearts. A**, Cardiac content of indirect lipid peroxidation marker 4-hydroxynonenal (4-HNE) protein adducts was evaluated by Western blot. β-actin was used as loading control. Quantitative analysis showing expression of 4-HNE (analyzed from 25kD to 100kD) relative to that of β-actin. **B,** 3-nitrotyrosine (3-NT) levels were measured by Western blot to estimate extent of oxidative stress and nitrative damage in cardiac tissue. β-actin used as loading control. Quantitative analysis showing expression of 3-NT (analyzed from 25kD to 100kD) relative to that of β-actin. All bar graphs are plotted as mean± SD. *P<0.05, **P<0.01 vs. HFHFD+IH group.

**Figure 6:**
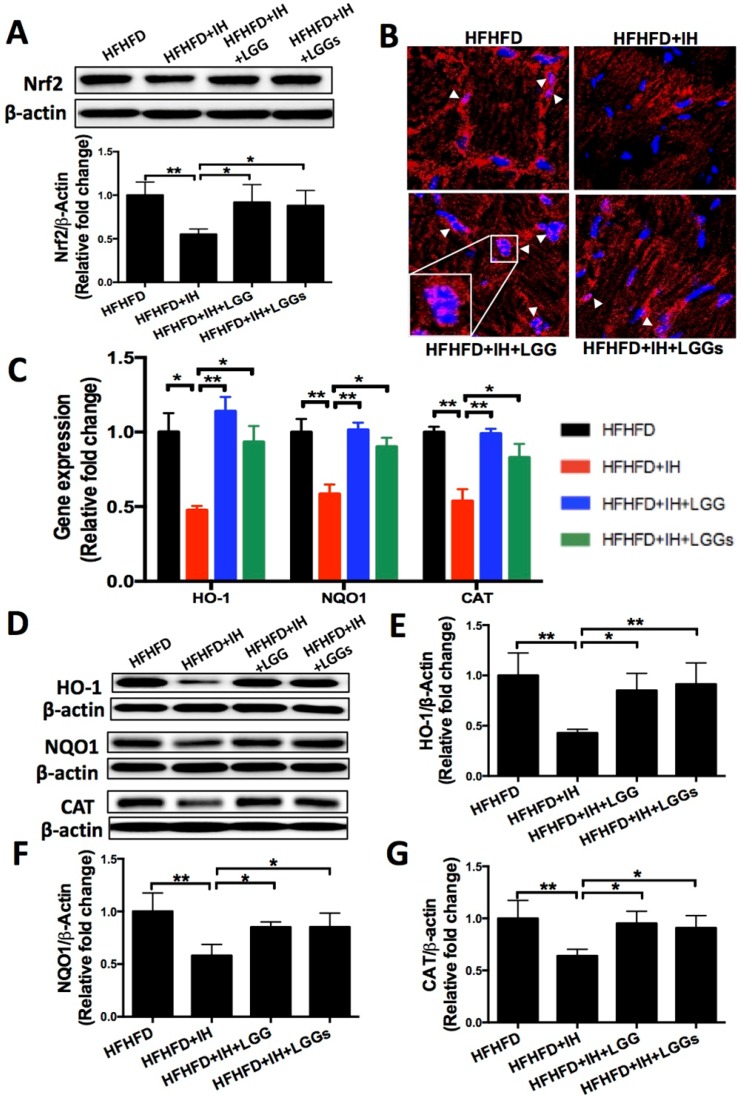
** The anti-oxidant effect of LGG and LGGs in IH mice hearts is Nrf2 dependent. A,** Nrf2 protein level (total protein was used) in heart tissues were determined by Western blot. β-actin was used as loading control. Quantitative analysis showing expression of Nrf2 relative to that of β-actin. **B**, Representative micrographs of cardiac frozen sections stained for Nrf2 (red) and DAPI (blue), showing restoration of nuclear accumulation of Nrf2 after treatment with LGG and LGGs. Arrows indicate overlapping IF signals. **C,** mRNA levels of Nrf2 downstream antioxidant effectors in IH-treated mice heart upon administration of LGG or LGGs, including HO-1, NQO1 and CAT. **D, E, F, G,** Western blot depicting protein levels of Nrf2 associated antioxidant markers, including NQO1, CAT, and HO-1. β-actin was used as loading control. Quantitative analysis showing expressions of NQO1, CAT, and HO-1 relative to that of β-actin. All bar graphs are plotted as mean± SD. *P<0.05, **P<0.01 vs. HFHFD+IH group.

IH can induce extra generation of intracellular reactive oxygen species (ROS), which leads to oxidative damage resulting in cardiac dysfunction 28. We therefore assessed the IH-induced oxidative damage under obese condition by immunoblot for 3-NT (a marker of protein nitration) and 4-HNE (a marker of lipid peroxidation). The results showed elevated expressions of 3-NT and 4-HNE under IH/obese conditions as compared to obese conditions alone (Fig. 5A, B). Treatment with LGG or LGGs showed a similar protective effect against IH-induced accumulation of 3-NT and 4-HNE proteins in obese mice (Fig. 5A, B). These results indicated that LGG and LGGs conferred heart protection against IH-associated oxidative stress and damage. In our previous study, IH was shown to reduce cardiac Nrf2 expression in mice, an effect that was reversed by sulforaphane treatment 11. We therefore investigated whether the antioxidant effect of LGG or LGGs was mediated via Nrf2. Consistent with previous studies, we found reduced expression of Nrf2 in IH/obese mouse hearts. After administration of LGG or LGGs, Nrf2 level was normalized to that in the normoxic group; there was no significant difference between the effect of LGG and LGGs on the Nrf2 levels in the hearts of IH/obese mice (Fig. 6A).

As Nrf2 functions only after its translocation to the nucleus, we next performed immunofluorescence for microscopic evidence of nuclear translocation of Nrf2. The results showed lesser accumulation of Nrf2 in the nuclei of cardiac cells in IH/obese mice as compared to that in obese mice; this phenomenon was reversed by administration of LGG or LGGs (Fig. 6B).

We then assessed the status of Nrf2 downstream anti-oxidant genes as an index of Nrf2 transcription activity. Consistent with the down-regulated Nrf2 expression and lesser nuclear accumulation, both mRNA (Fig. 6C) and protein (Fig. 6D-G) expressions of Nrf2 down-stream antioxidants NQO1, CAT, and HO-1 were significantly decreased in IH/obese mice compared to obese mice and reversed by LGG or LGGs. These findings suggest that LGG and LGGs are potential Nrf2 activators that may trigger anti-oxidative responses in heart tissues of obese mice in response to IH.

### Effects of LGG or LGGs on adiponectin receptors gene expression in IH-induced hearts

**Figure 7:**
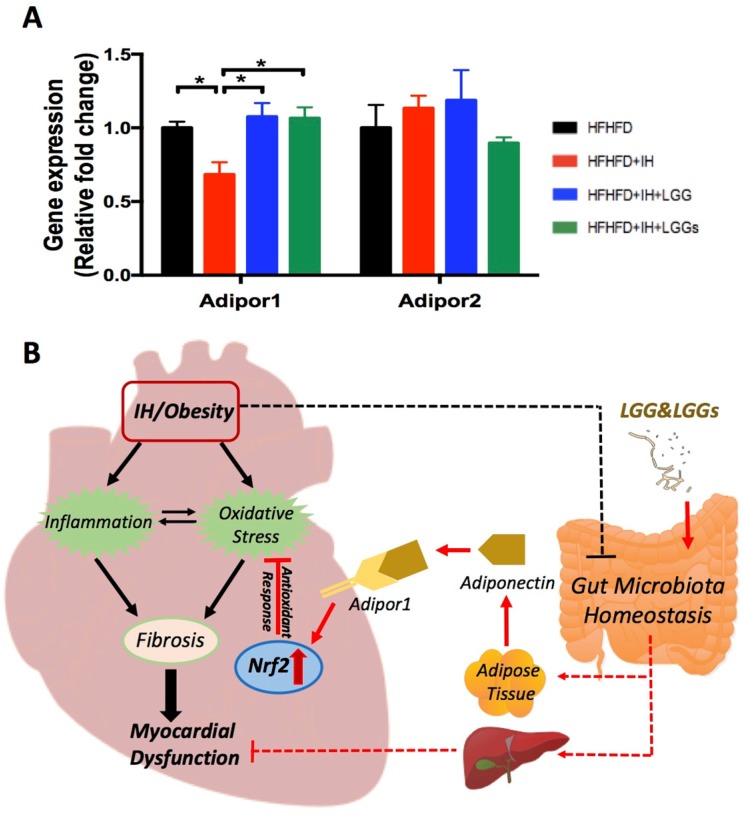
** Effects of LGG or LGGs on cardiac gene expression of Adipor1, and Adipor2 and global illustration. A,** mRNA levels of Adipor1 and Adipor2 in IH-treated mice heart upon administration of LGG or LGGs. All bar graphs are plotted as mean±SD. *P<0.05, **P<0.01 vs. HFHFD+IH group. **B,** Working hypothesis: IH disrupts myocardial function mainly via oxidative stress and inflammation, and also potentially modifies gut microbiota ecology. Administration of LGG or LGGs induces increased plasma levels of adiponectin. Adiponectin directly plays its role in heart by Adipor1 recognition and functions as an activator of cardiac Nrf2, eventually initiating antioxidative responses against IH related cardiomyopathy. Furthermore, a gut-liver-heart axis also exist to defend against IH associated myocardial dysfunction. The solid line represents the link supported by data from present study, whereas, the dash line represents the link supported by data from previous literatures. The black color represents effects caused by IH/obese, whereas, the red color represents effects resulted from LGG and LGGs.

A previous study reported that, compared to control mice, there is a marked increase of adiponectin in LGG-treated high fat diet-fed mice 29. Compared to IH conditions, our group demonstrated that treatment with LGGs significantly increased the serum levels of adiponectin in the present model (Liu Q, et al. Submitted). To assess the potential role of adiponectin in mediating cardiac protection, we profiled the expressions of genes that are known to act as receptors of adiponectin in cardiac tissues. Adiponectin exerts its function through two receptors, Adipor1 and Adipor2 30. We found that IH significantly downregulated cardiac Adipor1 expression, an effect that was reversed by treatment with LGG and LGGs. However, there was no significant change in Adipor2 expression, either under IH conditions or after treatment with LGG or LGGs (Fig. 7A). These results indicate that adiponectin may directly mediate the cardioprotective effect of LGG or LGGs against IH-induced cardiomyopathy. Additionally, Adipor1 possibly exerted a more important role than Adipor2 in mediating the cardioprotective effect of LGG or LGGs.

## Discussion

In the present study, we reported that OSA-induced IH contributes to the development of myocardial dysfunction in obese mice (although there was no significant cardiac hypertrophy) and is associated with increased inflammation and oxidative stress. Our data indicates that LGG and LGGs are potential activators of Nrf2 that may help preserve the antioxidant level and prevent cardiovascular complications in OSA. In addition, phosphorylation of NF-κB which mediates the inflammatory cascade effects was upregulated in IH/obese mice, but not in IH/obese mice treated either with LGG or LGGs. Collectively, these data demonstrate that oral supplementation with probiotic LGG or LGGs may improve myocardial function in obese mice exposed to IH by reducing inflammatory and oxidative effects along with preservation of Nrf2 expression and function (Fig. 7B).

IH induces inflammation and oxidative stress, leading to decreased expression of intestinal tight junction proteins 31, all of which are attributed to altered microbiota diversity and composition. Studies have shown that exposure to chronic IH decreases the genera *Lactobacillus* 32. In addition, bacteria-derived metabolites such as short-chain fatty acids are also influenced by chronic exposure to IH 33, 34 and these bacterial metabolites are associated with CVD. Animal studies involving administration of probiotics or probiotic metabolites support the beneficial effect of gut microbiome-targeted therapy on myocardial function. In the present study, IH exposure was shown to induce myocardial dysfunction, remodeling (such as wall thinning, enhanced chamber dilation, and reduced shortening fraction or ejection fraction), and marked increase in fibrosis. Moreover, the expressions of pro-fibrotic factors, fibronectin, collagen1A1, CTGF, and TGF-β were found enhanced in IH mice. However, the above adverse effects were prevented by supplementation with LGG or LGGs, which suggests a beneficial effect of probiotics and their metabolites on cardiovascular function in the IH model. In contrast, no significant between-group differences were observed with respect to myocyte size or expressions of embryo genes such as ANP and β-MHC. In a previous study, IH was shown to increase fasting serum levels of cholesterol, triglycerides (TGs), and phospholipids (PLs), as well as liver TG content in lean mice; however, obesity with baseline hypercholesterolemia was found to mask these IH-induced effects 35. Similarly, we speculate that the effect of IH on cardiomyocyte hypertrophy may have been masked in obese mice in the present study. The interaction between obesity and IH may alter the underlying mechanisms of cardiac remodeling in OSA.

IH-induced oxidative stress is mediated by either overproduction of ROS or weakening of the antioxidant defense system. Probiotics were shown to attenuate oxidative stress in obese rats as evidenced by decreased cardiac expressions of MDA and 4-HNE; these findings suggested that probiotic therapy ameliorated oxidative damage and improved cardiac mitochondrial respiratory complex functions^36^. Some studies have evaluated the anti-oxidative ability of cell-free probiotic supernatants. For instance, Shen et al. demonstrated strong antioxidant activity of cell free supernatant of *Bifidobacterium animalis* 01 in a mice model 37. In another study, probiotic metabolites in cell-free supernatant exhibited antioxidant properties by acting on PMA-stimulated human neutrophils 38. Consistently, findings from our present study show that IH-induced oxidative stress leads to protein damage and myocardial lipid peroxidation as supported by increased expressions of 3-NT and 4-HNE; this phenomenon was prevented by administration of LGG or LGGs.

Nrf2 is a well characterized redox-sensitive transcription factor. Activation of Nrf2 leads to upregulation of antioxidant defense genes, which play a key role in orchestrating antioxidant defenses and in maintaining redox homeostasis. Similarly, our findings indicate that the anti-oxidant effects of both LGG and LGGs against IH-induced cardiomyopathy were mediated via activation of Nrf2 and its target genes (such as *HO-1, NQO1*, and *CAT*). In another study, commensal lactobacilli were shown to activate responsive Nrf2 pathway, which induced a protective effect against environmental stress in lumen epithelium 39. However, few studies have evaluated these protective properties and the potential pathways of cell‐free probiotic cultures. Only the culture filtrate of *Lactobacillus helveticus* NS8 was shown to effectively protect the skin against chronic UVB‐induced oxidative damage and melanogenesis, an effect that was mediated via modulation of Nrf2 activity 40. All these findings highlight an antioxidative effect of LGG and LGGs via Nrf2 and its target genes that help redress redox metabolic imbalance in OSA related cardiomyopathy.

ROS and lipid peroxidation products act as chemo-attractants and signaling molecules, which contribute to the recruitment and activation of inflammatory cells; this in turn produces more oxidants and eventually induces a vicious cycle 41. Fibrosis may represent the final stage of a sustained inflammatory state, where fibrosis is triggered by chemokines, cytokines, and growth factors released by immune cells 42. The probiotic Lactobacillus reuteri GMNL-263 was shown to attenuate myocardial fibrosis and improve myocardial function in hamsters 43. To the best of our knowledge, most previous studies have focused on the immunomodulatory effects of probiotics; however, few studies have investigated the anti-inflammatory activity of cell-free supernatant of probiotics. In our previous study, LGGs were shown to improve the intestinal barrier and attenuate inflammation in alcohol-induced liver injury 23. There is a study has demonstrated the anti-inflammatory effects of supernatant from LGG in PMA (phorbol myristate acetate)-differentiated THP-1 cells. The results suggest anti-inflammatory properties of the supernatant from LGG 44. Our data support these studies in that supplementation of LGG or LGGs reduced NF-κB inflammatory cascades, as shown by decreased phosphorylated NF-κB, TNF-α, IL-1β, and PAI-1. Thus, the cardio-protective effect of LGG and LGGs against IH-induced cardiac inflammation could be due to the decreased expressions of cytokines and mediators.

The underlying mechanisms of the favorable effects of LGG or LGGs on cardiovascular system remain unclear. Adiponectin (an adipocyte-derived protein) in plasma acts as the predominant ligand for cardiac adiponectin receptors, exhibiting a cardioprotective role 45. Consistent with this, we found that the IH-induced downregulation of cardiac Adipor1 gene expression was restored by treatment with LGG or LGGs, without any significant change in Adipor2. Additionally, adiponectin supplementation was earlier shown to attenuate cardiac dysfunction by concomitantly restoring Nrf2 activation in hyperglycemia-induced cardiomyopathy 46. We hypothesize that the cardioprotective effect of LGG or LGGs in IH-exposed hearts is indirectly mediated by upregulation of adiponectin production, while adiponectin exerts a direct cardioprotective effect in conjunction with Adipor1; this results in activation of Nrf2 to trigger antioxidative defenses that protects the heart from IH-induced oxidative damage. Additionally, since LGG and LGGs were shown to protect against alcohol induced hepatic injury in our previous studies^23^, ^47^, as well as numerous evidence highlights the pivotal role of liver-heart crosstalk in CVD^48^, ^49^, we proposed a hypothesis that LGG and LGGs may lead to a gut-liver-heart axis formation and exhibit protective effects on heart (Fig. 7B).

There are several limitations in this study: (1) Only male mice were enrolled in this study. Several studies have demonstrated that sex hormones are associated with significant differences in the gut microbiota composition 50, and sex steroids attenuate many adverse effects of chronic hypoxia 51. (2) Our results only apply to obese mice with IH and cannot be generalized to healthy populations or those with other chronic illnesses. Obesity and OSA may have synergistic effects on the progression and severity of CVD. However, due to lack of mice with normal diet group, as well as a group with single treatment of IH, we cannot determine the effect of OSA or obesity alone on heart.

The realization that the LGG and its metabolites supernatant can reduce cardiac susceptibility provides a novel perspective and allows expansion of our approach to CVD research and therapeutic opportunities. Future studies should identify the mechanism by which gut microbiota contributes to CVD. The objectives should include defining the chemical mediators and both microorganism-microorganism and microorganism-host interactions within the complex microbiota ecosystem. Much work remains in order to understand the host receptors that perpetuate the signaling processes and lead to altered host cardiometabolism. Nrf2 knockout mice may serve as useful models of enhanced disease susceptibility. Thus, studies on Nrf2 knockout mice may help establish the role of these molecules in mediating the cardioprotective effects of intestinal microbiota. Moreover, exosomes in systemic circulation derived from intestine or liver may mediate the protective effects in multiple organs. Further studies are needed to investigate whether exosomes from digestive organs may mediate the cardiometabolic benefits.

In conclusion, our results suggest that supplementation with probiotics or probiotic secreted bioactive molecules might be an effective method to reduce CVD risk. Our study may help unravel the relationship of LGG and LGGs with improved myocardial function, as well as with decreased oxidative stress and inflammation. The molecular mechanisms of the antioxidant properties may involve activation of cardiac Nrf2. Overall, our results indicate that gut microbiota may represent a promising therapeutic target against cardiovascular complications in obese patients with OSA.

## Figures and Tables

**Table 1:** Echocardiographic parameters of HFHFD mice with IH.

	*HFHFD*	*HFHFD+IH*	*HFHFD+IH+LGG*	*HFHFD+IH+LGGs*
IVS, d (mm)	0.63±0.04	0.65±0.02	0.60±0.02	0.64±0.05
LVID, d (mm)	4.25±0.08	4.46±0.35	4.26±0.23	4.29±0.19
LVPW, d (mm)	0.77±0.02	0.75±0.03	0.68±0.09	0.74±0.04
IVS,s (mm)	1.02±0.06	1.01±0.06	0.94±0.08	0.97±0.15
LVID,s (mm)	2.68±0.06^*^	3.06±0.19	2.60±0.16^**^	2.73±0.21
LVPW,s (mm)	1.07±0.05	1.06±0.03	1.08±0.10	1.05±0.06
LV Vol, d (µL)	80.93±3.68	90.99±15.96	81.48±10.46	82.92±8.40
LV Vol, s (µL)	26.53±1.36^*^	36.98±5.55	24.67±3.67^**^	27.91±5.48
EF (%)	67.20±1.46^*^	59.05±3.94	69.76±1.46^**^	66.36±5.27^*^
FS (%)	36.97±1.17^*^	31.18±2.89	39.01±1.18^**^	36.46±3.96
LV mass (mg)	108.70±5.38	117.70±18.67	97.47±11.05	109.20±9.24

Data are presented as mean± SD. IVS, d: end-diastolic interventricular septum; IVS, s: end-systolic interventricular septum; LVID, d: left ventricular end-diastolic diameter; LVID, s: left ventricular end-systolic diameter; LVPW, d: left ventricular end-diastolic posterior wall; LVPW, s: left ventricular end-systolic posterior wall; LV Vol, d: left ventricular end-diastolic volume; LV Vol, s: left ventricular end-systolic volume; EF: ejection fraction; FS: fractional shortening; LV mass: left ventricular mass. *P<0.05, **P<0.01 vs. HFHFD+IH group

## Abbreviations

OSA: obstructive sleep apnea
IH: intermittent hypoxia
HFHFD: high-fat high-fructose diet
LGG: Lactobacillus rhamnosus GG
LGGs: Lactobacillus rhamnosus GG cell-free supernatant
Nrf2: nuclear factor erythroid 2-related factor 2
Keap1: Kelch-like ECH-associated protein 1
ARE: antioxidant response element
IVS, d: end-diastolic interventricular septum
IVS, s: end-systolic interventricular septum
LVID, d: left ventricular end-diastolic diameter
LVID, s: left ventricular end-systolic diameter
LVPW, d: left ventricular end-diastolic posterior wall
LVPW, s: left ventricular end-systolic posterior wall
LV Vol, d: left ventricular end-diastolic volume
LV Vol, s: left ventricular end-systolic volume
EF: ejection fraction
FS: fractional shortening
LV mass: left ventricular mass
FN: fibronectin
CTGF: connective tissue growth factor
TGF-β: transforming growth factor beta
WGA: wheat germ agglutinin
ANP: atrial natriuretic peptide
β-MHC: myosin heavy chain beta
p- NF-κB: phosphorylated nuclear factor kappa-light-chain-enhancer of activated B cells
t- NF-κB: total nuclear factor kappa-light-chain-enhancer of activated B cells
TNF-α: tumor necrosis factor-α
IL-1β: Interleukin-1β
PAI-1: plasminogen activator inhibitor-1
4-HNE: 4-hydroxynonenal
3-NT: 3-nitrotyrosine
NQO-1: NAD(P)H Quinone Dehydrogenase 1
CAT: catalase
HO-1: heme oxygenase 1
ROS: reactive oxygen species
Adipor1: adiponectin receptor 1
Adipor2: adiponectin receptor 2
SD: standard deviation
